# Risk of Ischemic Stroke, Hemorrhagic Stroke, Bleeding, and Death in Patients Switching from Vitamin K Antagonist to Dabigatran after an Ablation

**DOI:** 10.1371/journal.pone.0161768

**Published:** 2016-08-25

**Authors:** Jannik Langtved Pallisgaard, Gunnar Hilmar Gislason, Christian Torp-Pedersen, Christina Ji-Young Lee, Caroline Sindet-Pedersen, Laila Staerk, Jonas Bjerring Olesen, Tommi Bo Lindhardt

**Affiliations:** 1 Department of Cardiology, Copenhagen University Hospital Herlev and Gentofte, Copenhagen, Denmark; 2 Faculty of Health and Medical Sciences, University of Copenhagen, Copenhagen, Denmark; 3 The Danish Heart Foundation, Copenhagen, Denmark; 4 The National Institute of Public Health, University of Southern Denmark, Odense, Denmark; 5 Institute of Health, Science and Technology, Aalborg University, Aalborg, Denmark; University of Bologna, ITALY

## Abstract

**Background:**

Safety regarding switching from vitamin K antagonist (VKA) to dabigatran therapy in post-ablation patients has never been investigated and safety data for this is urgently needed. The objective of this study was to examine if switch from VKA to dabigatran increased the risk of stroke, bleeding, and death in patients after ablation for atrial fibrillation.

**Methods:**

Through the Danish nationwide registries, patients with non-valvular atrial fibrillation undergoing ablation were identified, in the period between August 22nd 2011 and December 31st 2015. The risk of ischemic stroke, hemorrhagic stroke, bleeding, and death, related to switching from VKA to dabigatran was examined using a multivariable Poisson regression model, where Incidence rate ratios (IRR) were estimated using VKA as reference.

**Results:**

In total, 4,236 patients were included in the study cohort. The minority (n = 470, 11%) switched to dabigatran in the follow up period leaving the majority (n = 3,766, 89%) in VKA treatment. The patients in the dabigatran group were older, were more often males, and had higher CHA2DS2-VASc, and HAS-BLED scores. The incident rates of bleeding and death were almost twice as high in the dabigatran group compared with the VKA group. When adjusting for the individual components included in the CHA2DS2-VASc and HAS-BLED scores, the multivariable Poisson analyses yielded a non-significant IRR (95%CI) of 1.64 (0.72–3.75) for bleeding and of 1.41 (0.66–3.00) for death associated with the dabigatran group, compared to the VKA group. A significant increased risk of bleeding was found in the 110mg bid group with an IRR (95%CI) of 4.49(1.40–14.5).

**Conclusion:**

Shifting from VKA to dabigatran after ablation was associated with twice as high incidence of bleeding compared to the incidence in patients staying in VKA treatment. The only significant increased risk found in the adjusted analyses was for bleeding with 110mg bid dabigatran and not for 150mg bid. Since there was no dose-response for bleeding, the switch from VKA to dabigatran in itself was not a risk factor for bleeding.

## Introduction

In current guidelines, patients undergoing ablation for atrial fibrillation should receive systemic anticoagulation at a therapeutic level prior to the ablation procedure for a minimum of 3 weeks and a minimum of 2 months after the ablation.[1] The Danish guidelines recommend vitamin K antagonists (VKA) as only accepted anticoagulation therapy for ablation for atrial fibrillation.[2] Regardless of post-ablation atrial fibrillation status, patients with indication for anticoagulation therapy are advised to continue their anticoagulation therapy as if atrial fibrillation was still present.[1] In Denmark, dabigatran have since August 22^nd^ 2011 been available as an alternative to VKA in patients with non-valvular atrial fibrillation. Dabigatran is less challenging for patients and physicians, since coagulation monitoring and subsequent dose adjustments are not routinely required, no food restrictions apply and interactions with concomitant drugs are less relevant compared with VKA.[3,4] As a consequence some patients will prefer dabigatran treatment over VKA and switched from VKA to dabigatran after the ablation procedure. International guidelines recommend that a switch to dabigatran can be initiated once the INR is equal to or lower than 2.0.[5] These recommendations are derived from observations of the large phase III programs, in which about half of included patients were VKA experienced.[6] However, a shift from VKA to dabigatran could impose a risk of both thromboembolism and bleeding. The safety of switching from VKA to dabigatran therapy in post-ablation patients has never been investigated and safety data for this is urgently needed. The objective of this study was to examine if switch from VKA to dabigatran increased the risk of stroke, bleeding, and death in patients after ablation for atrial fibrillation.

## Method

In Denmark, all residents are at birth or immigration provided with a permanent and unique civil registration number that enables individual-level linkage between administrative registries.[7] The Danish National Patient Register holds information on all hospitals visits of both in and out patients in Denmark since 1978. Each hospitalization is at discharge coded with one primary and, if appropriate, one or more secondary diagnosis codes according to the International Classification of Diseases, the 8th revision (ICD-8) until 1994 and the 10th revision (ICD-10) thereafter.[8] All hospital procedures in Denmark have been registered since 1996 and coded according to the Nordic Classification of Surgical Procedures (NCSP) by The Nordic Medico-Statistical Committee.[8] The Danish Registry of Medicinal Product Statistics keeps records on all drug prescriptions dispensed from Danish pharmacies since 1995.[9] Each drug dispensing is registered according to an international classification of drugs, the Anatomical Therapeutic Chemical (ATC) system, as well as the date of dispensing, quantity dispensed, strength, formulation, and affiliation of the physician issuing the prescription. Prescriptions are partially reimbursed by the Danish health care system.

### Study cohort and follow up

All Danish patients with non-valvular atrial fibrillation undergoing ablation for atrial fibrillation between August 22^nd^ 2011 and December 31^st^ 2015 were identified. In order to avoid measuring post procedure complications patients were finally included 30 days after the ablation date, which was defined as the inclusion date. All patients who received an additional ablation, died or were not in VKA treatment at the inclusion day were excluded. All patients were allocated to either the VKA group for those who stayed in VKA treatment or the dabigatran group for those who shifted from VKA to dabigatran in the follow-up period. Patients were followed from inclusion date until the date of a study outcome, date of switch to apixaban or rivaroxaban, date of switch from dabigatran back to VKA, last day of anticoagulation treatment or the end of study March 1 2015, which ever came first. To avoid misclassifying small gaps between prescriptions as treatment discontinuation a grace period of 30 days between prescriptions was offered. Patients in the dabigatran group were identified as prior users if they had been on dabigatran within six months prior to ablation date, the rest of the patients were identified as de novo users.

### Comorbidities and concomitant medication

Comorbidities were identified using ICD-10 codes for: stroke, heart failure, cancer, acute coronary syndrome, ischemic heart disease, chronic obstructive pulmonary disease, chronic kidney disease, liver disease, alcohol abuse, bleeding, and vascular disease. Claimed drug prescriptions were used to identify, acetylsalicylic acid, ADP receptor inhibitors, RAS inhibitors, beta blockers, calcium channel blockers, amiodarone, digoxin, flecainide, spironolactone and NSAID use. Hypertension was defined from the use of at least two classes of antihypertensive drugs and diabetes was defined from the use of antidiabetic drugs. CHADS_2_, CHA_2_DS_2_-VASc and HAS-BLED scores were calculated from comorbidities and drug use. NCSP codes were used to identify prior Coronary artery bypass graft, and Percutaneous Coronary Intervention, ablation. (S1 Table).

### Study outcome

The study outcomes were hospital admissions with ischemic stroke, hemorrhagic stroke, bleeding, and all-cause mortality. These were investigated as both individual endpoints and as a composite of all individual endpoints (ischemic stroke, hemorrhagic stroke, bleeding, and all-cause mortality).

### Statistical methods

The demographic characteristics were described according to anticoagulation status on either the inclusion date (VKA group) or the day patients switch to dabigatran (dabigatran group). Categorical data was presented as counts with percentages, and statistical difference was tested using Chi-squared test and Fisher's exact test where appropriate. Continuous variables were presented as mean with standard deviations for normal distributed data, and as medians with interquartile range for non-normal distributed data, and statistical difference was tested using Student's t-test and Wilcoxon rank-sum test where appropriate. The study cohort characteristics were continuously updated with one-year splits on each calendar year, one-year age increase, and at dates of comorbidity onset. Incidence rates of all endpoints were calculated as number of events per 1,000 person years in both groups and the relative risk was calculated as incidence rate ratio (IRR) with 95% confidence intervals (95%CI) for dabigatran use with VKA as reference using a multivariable Poisson model adjusting for the individual components of the CHA_2_DS_2_-VASc and HAS-BLED scores. Additional analyses with the VKA group as reference were conducted including: subgroups of patients with a history of dabigatran use prior to ablation and de novo dabigatran users; a subgroup with maximum one year follow-up period after ablation; a subgroup with 110mg bid dabigatran use; and a subgroup with 150mg bid dabigatran use. Data management and statistical analyses were conducted using R statistics (R Core Team (2015). R: A language and environment for statistical computing. R Foundation for Statistical Computing, Vienna, Austria. URL http://www.R-project.org/.)

### Ethics approval

In Denmark, retrospective register studies do not require approval from the ethics committees. The Danish Data Protection Agency approved this study (ref. no.: 2007-58-0015/GEH-2014-016 I-Suite no.: 02734) and data were made available in an anonymized format such that specific individuals could not be identified.

## Results

**Fig 1:**
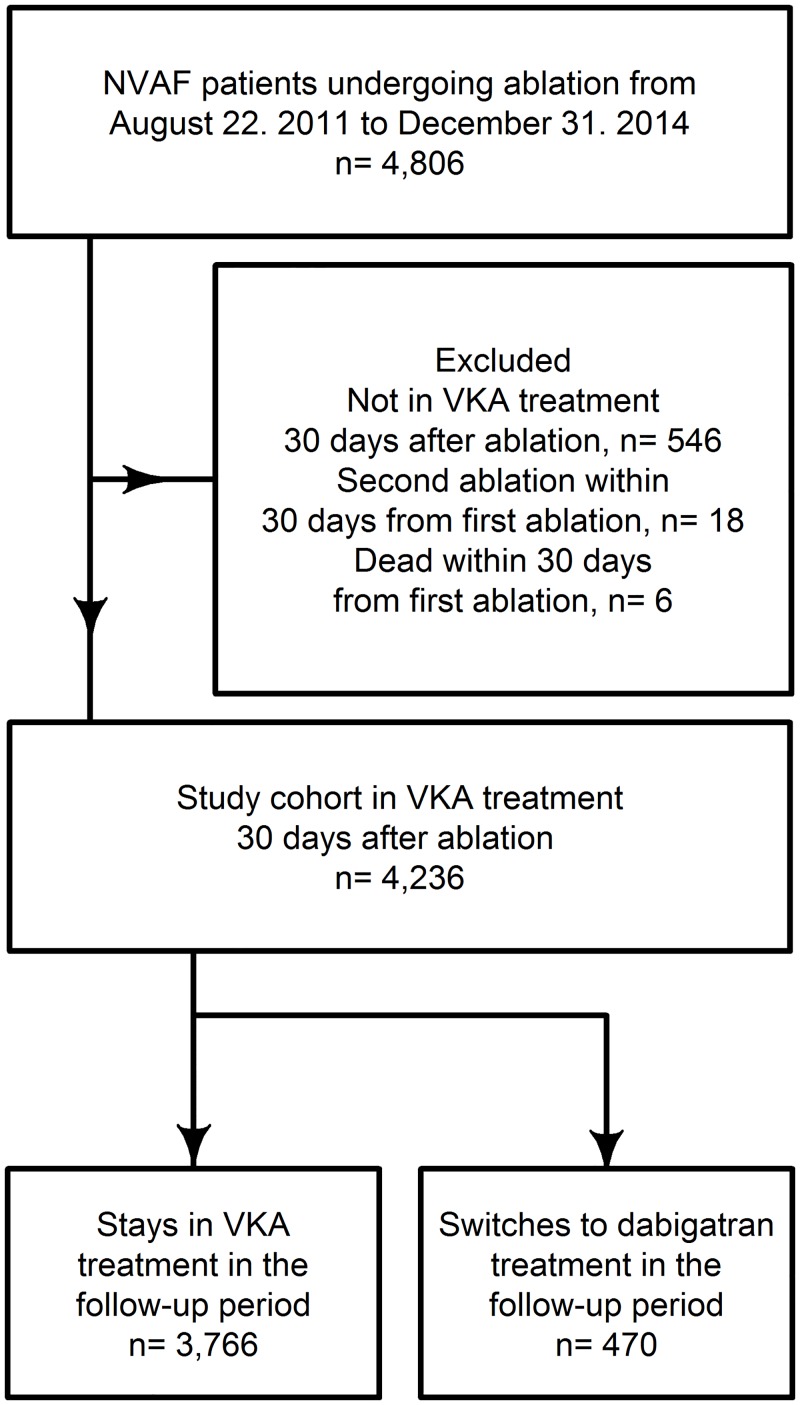
Flowchart of the study cohort. Legend: NVAF = non-valvular atrial fibrillation; VKA = Vitamin-K-antagonist

From the 4,806 in ablations in the inclusion period (August 22^nd^ 2011 and December 31^st^ 2015), 4,236 (88%) were in VKA treatment 30 days after the ablation date and included in the study cohort. The minority (n = 470, 11%) switched to dabigatran in the follow up period leaving the majority (n = 3,766, 89%) in VKA treatment (Fig 1). Patients in the dabigatran group were older, more often males, and had higher CHA_2_DS_2_-VASc and HAS-BLED scores (Table 1). The follow up median time was 310 days with interquartile range 178 to 420 days.

**Table 1:** Baseline characteristics of patients in the VKA group and the dabigatran group.

	VKA group	Dabigatran group	p
n	3,766	470	
Age (median [IQR])	62.9 [55.1, 68.8]	65.1 [58.0, 70.0]	<0.001
Age category, n (%)			<0.001
<65 years, n (%)	2,194 (58.3)	233 (49.6)	
65–75 years, n (%)	1,315 (34.9)	211 (44.9)	
>75 years, n (%)	257 (6.8)	26 (5.5)	
Men, n (%)	2,816 (74.8)	317 (67.4)	0.001
Ischemic stroke, n (%)	99 (2.6)	21 (4.5)	0.034
Hemorrhagic stroke, n (%)	13 (0.3)	6 (1.3)	0.013
Prior AMI or angina, n (%)	894 (23.7)	125 (26.6)	0.190
Coronary disease, n (%)	569 (15.1)	73 (15.5)	0.863
Atherosclerosis, n (%)	60 (1.6)	5 (1.1)	0.496
Cancer, n (%)	214 (5.7)	32 (6.8)	0.379
Liver, n (%)	57 (1.5)	7 (1.5)	1.000
Chronic Obstructive Pulmonary disease, n (%)	218 (5.8)	32 (6.8)	0.435
Heart Failure, n (%)	648 (17.2)	89 (18.9)	0.385
Chronic Kidney Disease, n (%)	111 (2.9)	6 (1.3)	0.036
Bleeding, n (%)	498 (13.2)	66 (14.0)	0.674
Hypertension, n (%)	1,683 (44.7)	226 (48.1)	0.178
Diabetes, n (%)	337 (8.9)	39 (8.3)	0.703
RAS Inhibitors, n (%)	1,683 (44.7)	225 (47.9)	0.208
Beta Blockers, n (%)	2,818 (74.8)	362 (77.0)	0.327
Amiodarone, n (%)	926 (24.6)	143 (30.4)	0.007
Calcium Channel Blockers, n (%)	869 (23.1)	117 (24.9)	0.411
Acetylsalicylic Acid, n (%)	692 (18.4)	86 (18.3)	1.000
NSAID, n (%)	345 (9.2)	36 (7.7)	0.324
ADP-Inhibitors, n (%)	110 (2.9)	5 (1.1)	0.029
Digoxin, n (%)	580 (15.4)	92 (19.6)	0.023
Flecainide, n (%)	544 (14.4)	68 (14.5)	1.000
Spironolactone, n (%)	236 (6.3)	38 (8.1)	0.158
Prior Coronary Angiograms, n (%)	248 (6.6)	26 (5.5)	0.438
Prior Bypass Surgery, n (%)	103 (2.7)	8 (1.7)	0.243
CHA_2_DS_2_-VASc (median [IQR])	1 [0, 2]	2 [1, 2]	<0.001
CHA_2_DS_2_-VASc groups, n (%)			<0.001
0	947 (25.1)	65 (13.8)	
1	1,071 (28.4)	143 (30.4)	
>1	1,748 (46.4)	262 (55.7)	
HAS-BLED (median [IQR])	1 [0, 2]	1 [1, 2]	0.011
HAS-BLED groups, n (%)			0.001
0	946 (25.1)	80 (17.0)	
1	1,307 (34.7)	179 (38.1)	
>1	1,513 (40.2)	211 (44.9)	

**Fig 2:**
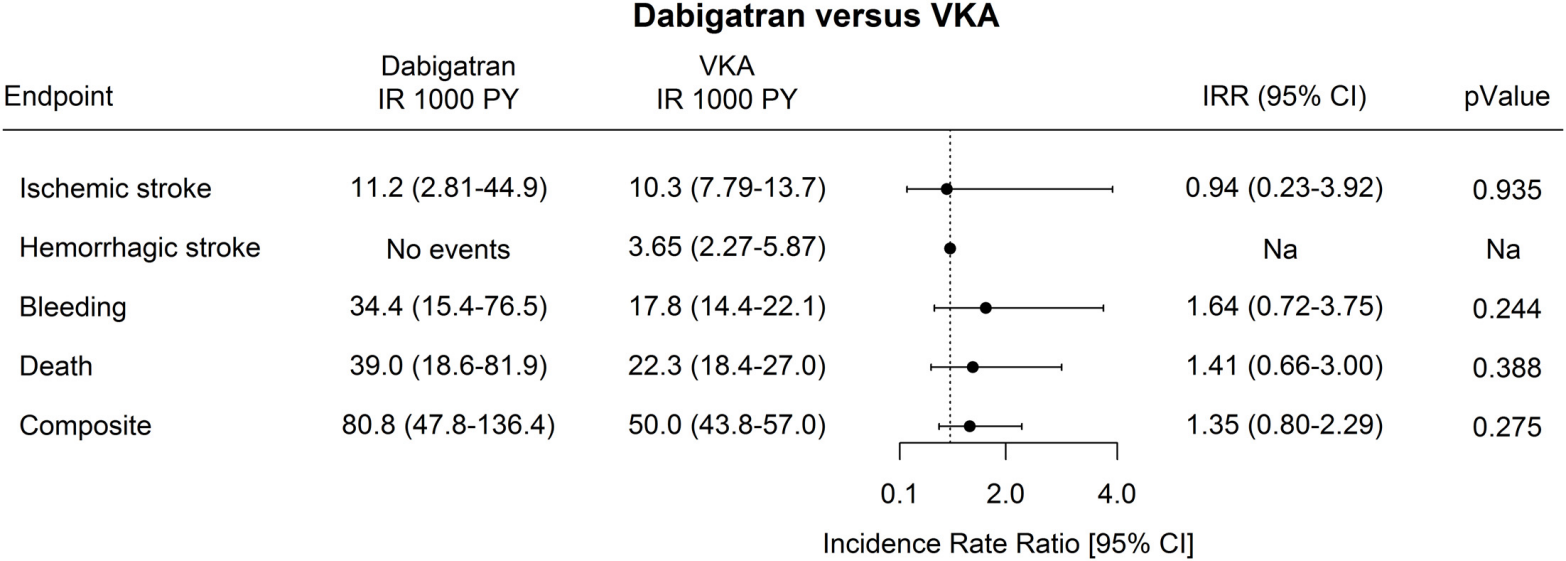
Poisson Regression. Incidence rate ratios of ischemic or hemorrhagic stroke, myocardial infarction, bleeding, death, or a composite of these. Legend: Number of dabigatran users n = 470. Composite is a combined endpoint of ischemic and hemorrhagic stroke, bleeding, and death. All patients with prior events of the investigated endpoint were excluded in the analysis. Models were adjusted for: CHA2DS2-VASc and HAS-BLED components. IR = Incidence Rate, PY = Person years, CI = Confidence Intervals

The incidence rates of ischemic stroke were equal between the two groups with am incidence rate per 1,000 person years of 11.2 (2.81–44.9) in the dabigatran group and 10.3 (7.79–13.7) in the VKA group. No statistical difference found between the two groups. Hemorrhagic stroke events were very rare with no events found with dabigatran, and an incidence rate per 1,000 person years of 3.65 in the VKA group. The incident rates of bleeding and death were almost twice as high in the dabigatran group compared with the VKA group. When adjusting for the individual risk factors included in the CHA_2_DS_2_-VASc and HAS-BLED scores, the multivariable analyses yielded an IRR (95%CI) of 1.64 (0.72–3.75) for bleeding and of 1.41 (0.66–3.00) for death associated with the dabigatran group, compared to the VKA group. The incidence rates of a composite event were almost twice as high in the dabigatran group compared with the VKA group. No significant difference found between the two groups with an IRR (95%CI) of 1.35 (0.80–2.29) (Fig 2).

**Fig 3:**
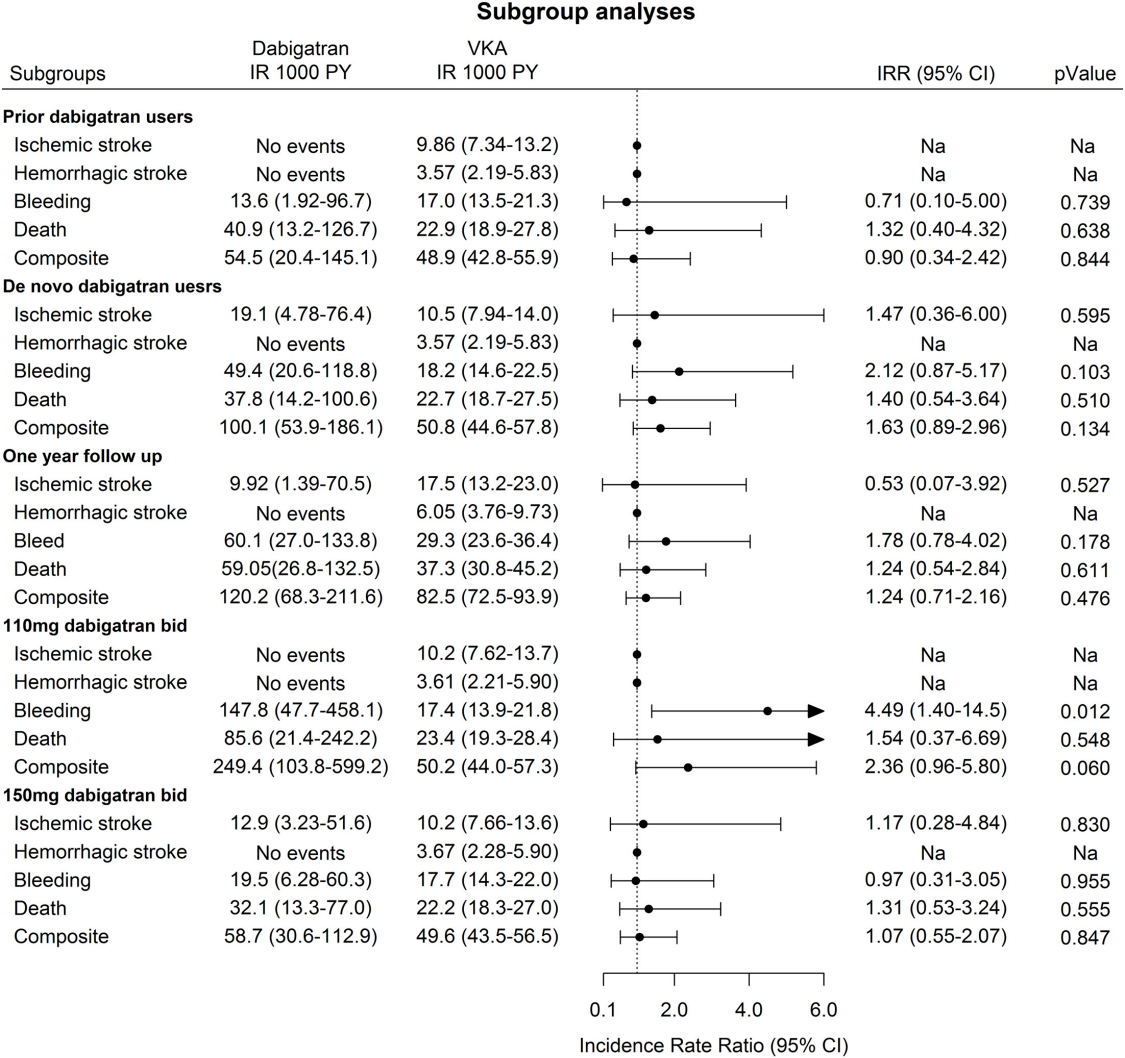
Poisson Regression. Incidence rate ratios of ischemic or hemorrhagic stroke, myocardial infraction, bleeding, death, and a composite of these. Legend: Prior dabigatran users defined as dabigatran use prior to ablation. De novo dabigatran users defined as no dabigatran use prior to ablation. One year follow up defined as follow up time for a maximum time of 1 year after ablation. 110 mg dabigatran bid defined as prescribed with 110mg dabigatran at switch date. 150mg dabigatran bid defined as prescribed with 150mg dabigatran at switch date. Composite is a combined endpoint of ischemic and hemorrhagic stroke, bleeding, and death. All patients with prior events of the investigated endpoint were excluded in the analysis. Models were adjusted for: components in CHA2DS2-VASc and HAS-BLED

Patients with prior dabigatran use were older and had higher CHA_2_DS_2_-VASc and HAS-BLED score compared with de novo users of dabigatran (Table 2). The incidence of the composite event was almost equal between the two groups. When adjusting for the individual risk factors included in the CHA_2_DS_2_-VASc and HAS-BLED scores, no significant difference in risk of the composite event was found between the dabigatran group and the VKA group with an IRR (95%CI) 0.90 (0.34–2.42) (Fig 3). In the subgroup analyses, including only de novo dabigatran users the incidence rates were twice as high in the dabigatran group compared to the VKA group and in the adjusted analysis an non-significant difference of the composite event in the dabigatran group compared to the VKA group with an IRR (95%CI) of 1.63 (0.89–2.96) (Fig 3).

**Table 2:** Baseline characteristics of patients with de novo use of dabigatran and prior users of dabigatran. Prior users if they had been on dabigatran within six months prior to ablation date, the rest of the patients were identified as de novo users.

	De novo users	Prior users	p
N	253	217	
Age (median [IQR])	63.8 [57.8, 68.7]	66.8 [58.5, 70.6]	0.024
Age category, n (%)			0.001
<65 years, n (%)	144 (56.9)	89 (41.0)	
65–75 years, n (%)	94 (37.2)	117 (53.9)	
>75 years, n (%)	15 (5.9)	11 (5.1)	
Men, n (%)	174 (68.8)	143 (65.9)	0.572
Ischemic stroke, n (%)	10 (4.0)	11 (5.1)	0.719
Hemorrhagic stroke, n (%)	<3	4 (1.8)	0.548
Prior AMI or angina, n (%)	72 (28.5)	53 (24.4)	0.378
Coronary disease, n (%)	40 (15.8)	33 (15.2)	0.958
Atherosclerosis, n (%)	<3	4 (1.8)	0.283
Cancer, n (%)	13 (5.1)	19 (8.8)	0.171
Liver, n (%)	4 (1.6)	3 (1.4)	1.000
Chronic Obstructive Pulmonary disease, n (%)	22 (8.7)	10 (4.6)	0.116
Heart Failure, n (%)	43 (17.0)	46 (21.2)	0.298
Chronic Kidney Disease, n (%)	5 (2.0)	<3	0.224
Bleeding, n (%)	42 (16.6)	24 (11.1)	0.112
Hypertension, n (%)	115 (45.5)	111 (51.2)	0.254
Diabetes, n (%)	22 (8.7)	17 (7.8)	0.865
RAS Inhibitors, n (%)	112 (44.3)	113 (52.1)	0.110
Beta Blockers, n (%)	188 (74.3)	174 (80.2)	0.162
Amiodarone, n (%)	71 (28.1)	72 (33.2)	0.271
Calcium Channel Blockers, n (%)	61 (24.1)	56 (25.8)	0.751
Acetylsalicylic Acid, n (%)	58 (22.9)	28 (12.9)	0.007
NSAID, n (%)	18 (7.1)	18 (8.3)	0.760
ADP-Inhibitors, n (%)	4 (1.6)	<3	0.466
Digoxin, n (%)	56 (22.1)	36 (16.6)	0.163
Flecainide, n (%)	39 (15.4)	29 (13.4)	0.618
Spironolactone, n (%)	22 (8.7)	16 (7.4)	0.723
Prior Coronary Angiogram, n (%)	14 (5.5)	12 (5.5)	1.000
Prior Bypass Surgery, n (%)	6 (2.4)	<3	0.393
CHA_2_DS_2_-VASc (median [IQR])	1 [1, 2]	2 [1, 2]	0.002
CHA_2_DS_2_-VASc groups, n (%)			<0.001
0	48 (19.0)	17 (7.8)	
1	82 (32.4)	61 (28.1)	
>1	123 (48.6)	139 (64.1)	
HAS-BLED (median [IQR])	1 [1, 2]	1[1, 2]	0.458
HAS-BLED groups, n (%)			0.031
0	53 (20.9)	27 (12.4)	
1	87 (34.4)	92 (42.4)	
>1	113 (44.7)	98 (45.2)	

In the subgroup analyses with a maximum of one year follow up period the results were largely the same as in the main analyses (Fig 2). Only a very few patients (n = 57) started in 110mg bid dabigatran, but despite of this a significant increased risk of bleeding was found in this group with an IRR (95%CI) of 4.49(1.40–14.5). There was no risk of any events in 150mg bid dabigatran group.

## Discussion

The main result of our study was that patients with non-valvular atrial fibrillation shifting from VKA to dabigatran after an ablation had approximately twice as high incidence rates of bleeding, and death compared to patients continuing in VKA treatment. The higher incidence was mainly due to increased incidence rates of bleeding and death found in the de novo users of dabigatran and in the patients treated with 110 mg bid dabigatran. These patients were in general older and had more co-morbidities. Amiodarone increases plasma levels of both dabigatran and VKA and therefore concomitant treatment with amiodarone and dabigatran should be carefully evaluated in patients at high risk of bleeding. Significantly more patients received amiodarone in the dabigatran group compared with the VKA group (926 (24.6) versus 143 (30.4), p = 0.007). In the multivariable analyses no significant differences were observed between the patients continued on VKAs and the patients shifted to dabigatran.

Observational studies investigating risks associated with switch from VKAs to dabigatran are scarce and to the best of our knowledge, none exists with a study cohort of post ablation patients. Sørensen et al. (2013) investigated thromboembolic events and risk of bleeding in atrial fibrillation patients switching from VKA to dabigatran. Subgroups of both 110mg bid and 150mg bid dabigatran were investigated.[10] Similar to our results an increased risk of bleeding was found only in the 110 mg bid group and not in the dabigatran 150 mg bid group. This could suggest that the switch from VKA to dabigatran in itself was not a risk factor for bleeding, since the shift would be an equal risk factor in both groups. The increased risk could therefore be due to other predominant risk factors in the 110mg bid group, e.g. age ≥80 years and impaired renal function.[11] It would be unlikely that a lower dose dabigatran (110mg bid) would increase risk of bleeding compared to the high dose (150mg bid), since this would implicate a reciprocal dose-response bleeding risk. It would be unlikely that a lower dose dabigatran (110mg bid) would increase risk of bleeding compared to the high dose (150mg bid), since this would implicate a reciprocal dose-response bleeding risk.

Dissimilar to our results the risk of stroke was increased among patients switching to dabigatran. There was no indication of an increased risk of stroke in our study. Larsen et al. (2014) also investigated risk of bleeding in atrial fibrillation patients switching from VKA to dabigatran.[12] They found no risk of bleeding for switchers to dabigatran 150 mg bid, HR (95%CI) of 0.80 (0.62–1.03) and 110 mg bid HR (95%CI) of 1.12 (0.90–1.41). Similar to our findings a no significant bleeding risk was found in switchers to dabigatran 150 mg bid, but when switching to 110mg bid an increased risk of bleeding was found in our study. The main difference between the our and the study by Larsen et al. is our study cohort was a post ablation cohort. Bouillon et al. (2015) investigated the risk of bleeding in a case control study matching patients switching from treatment with a VKA to a non-vitamin K antagonist oral anticoagulant (NOAC) with patients who continued VKA treatment. No significant difference in bleeding risk was found when patients switched from VKA to dabigatran (HR 0.78, 95% CI 0.54–1.09) [13].

In the subgroup of de novo and prior dabigatran users in our study, no significant difference in the risk of a composite event was found with either the de novo dabigatran users, or the prior dabigatran users. The subgroup analyses of patients in the 110mg bid dabigatran group relied on a very low number of patients (n = 57). The results from these subgroup analyses should therefore be interpreted with large caution. On the other hand the IRR (95%CI) for a composite event was 2.36 (0.96–5.80) in the 110mg bid group compared to the IRR (95%CI) of 1.07 (0.55–2.07) in the 150mg bid group. This suggests that the composite events in the dabigatran group originated from the 110mg bid subgroup and not the 150mg bid dabigatran group.

### Strengths and limitations

One major limitation of this study is inherent in the observational nature of our study where confounders and risk factors may have influenced our results. These unmeasured risk factors could very well have been predominant in the de novo subgroup and in the 110mg bid subgroup. The strength of this study is the completeness of data with a nationwide unselected cohort of ablation patients. The register data used have previously been validated [14–17]. Immortal time bias was avoided in the dabigatran group, because all patients were at risk from the inclusion date and during follow up.

## Conclusion

Shifting to dabigatran after ablation was associated with twice as high incidence of bleeding compared to patients continuing VKA treatment. A significantly increased risk of bleeding was found in in patients treated with 110mg bid dabigatran, but not for 150mg bid. Since there was no dose-response for bleeding the switch from VKA to dabigatran was in itself, not a risk factor for bleeding.

## Supporting Information

S1 Table Appendix. The International Classification of Diseases (ICD) codes and Anatomical Therapeutic Chemical (ATC) system codes.
(DOCX)
