# Supplementary material for: Risk of Ischemic Stroke, Hemorrhagic Stroke, Bleeding, and Death in Patients Switching from Vitamin K Antagonist to Dabigatran after an Ablation
Source: PLoS One. 2016 Aug 25;11(8):e0161768. doi: 10.1371/journal.pone.0161768 (PMC4999147; doi:10.1371/journal.pone.0161768)
Supplement: S1 Table — The International Classification of Diseases (ICD) codes and Anatomical Therapeutic Chemical (ATC) system codes. (DOCX) [file pone.0161768.s001.docx]

| S1 Table. Appendix. The International Classification of Diseases (ICD) codes and Anatomical Therapeutic Chemical (ATC) system codes. | |
| --- | --- |
| ICD-8 and ICD-10 | |
| Atrial fibrillation | I48 |
| Rheumatic Heart Valve Disease | DI05, DI06, DZ952, DZ954, DI080A, DI081A, DI082A, DI083A |
| Heart failure | I50, I500, I501, I501A, I501B, I501C, I501D, I503, I508, I508A, I509, I509A, I509B, DJ819 |
| Stroke | I60, I600, I601, I602, I603, I604, I605, I606, I606A, I606B, I606C, I606D, I607, I607A, I608, I609, I609A, I61, I610, I610A, I611, I611A, I611B, I612, I613, I614, I615, I616, I618, I619, I62, I620, I621, I629, I63, I630, I631, I632, I633, I634, I634A, I635, I636, I638, I639, I64, I649 |
| Chronic kidney disease | DE102, DE112, DE132, DE142, I12, I120, I129, I129A, N03, N030, N031, N032, N033, N034, N035, N035A, N035C, N036, N037, N038, N038A, N038B, N039, N04, N040, N041, N042, N043, N044, N045, N045A, N045C, N046, N047, N048, N048A, N048B, N049, N05, N050, N051, N052, N053, N054, N055, N055B, N055C, N056, N057, N058, N058A, N058B, N059, N06, N060, N061, N062, N063, N064, N065, N065C, N066, N067, N068, N068A, N068B, N069, N07, N070, N071, N072, N073, N074, N075, N075A, N075C, N076, N077, N078, N079, N08, N080, N080A, N080B, N080E, N081, N081A, N081B, N082, N082A, N082B, N082D, N082E, N083, N084, N084B, N084C, N084E, N085, N085A, N085B, N085C, N085D, N085E, N088, N088A, N11, N110, N110A, N111, N111A, N111B, N112, N118, N118A, N118B, N118C, N118D, N119, N13, N130, N131, N131A, N132, N132A, N132B, N133, N133A, N134, N135, N135A, N135B, N135C, N136, N136A, N136B, N136C, N136D, N136E, N137, N138, N138A, N139, N139A, N139B, N139C, N14, N140, N140A, N141, N142, N143, N144, N18, N180, N181, N182, N183, N184, N185, N188, N188A, N188B, N189, N19, N199, N25, N250, N250B, N250C, N250D, N251, N258, N258A, N258B, N258C, N259, N26, N269, N27, N270, N271, N279, N28, N280, N280A, N280B, N280C, N280D, N281, N288, N288B, N288D, N288E, N288F, N288G, N288H, N288J, N289, N289A, N29, N290, N291, N298, N298A, N391, Q61, Q610, Q610A, Q610B, Q611, Q612, Q613, Q614, Q615, Q618, Q618A, Q619, Q619A |
| Liver disease | K70, K700, K701, K702, K703, K703A, K704, K704A, K704B, K704C, K704D, K709, K71, K710, K710A, K711, K711A, K711B, K711C, K711D, K712, K712A, K712B, K713, K713A, K713B, K714, K714B, K715, K715A, K715C, K715D, K716, K716A, K716B, K717, K717A, K717B, K718, K719, K72, K720, K720A, K720C, K720D, K720E, K720F, K721, K729, K729A, K73, K730, K731, K732, K732A, K732B, K732C, K732D, K732E, K732F, K732G, K738, K739, K74, K740, K740B, K741, K742, K743, K743A, K744, K745, K746, K746A, K746B, K746C, K746D, K746E, K746F, K746G, K746H, K75, K750, K750A, K750B, K750C, K750D, K751, K751A, K752, K753, K754, K758, K759, K759A, K76, K760, K760A, K760B, K760C, K761, K761A, K761B, K762, K763, K764, K764A, K765, K766, K766A, K766B, K767, K768, K768A, K768B, K768C, K768E, K769, K77, K770, K770A, K770D, K778, K778A, K778B |
| Vascular disease | I803, I269, I269A , I802 , I829 , I809 , I260, I803F, I803E, I801, I829B, I802B , I828, I801B, I803D, I822, I803B, I803C, I260A, I803A, I802A, I809B, I823, I822B, I829A, I26, I821, I809A, I801A, I823B, I822A, I823A, I743, I742, I744, I743A, I749, I743B, I742A, I745, I740, I748, I742B, I741, I744B, I744A, I74, I740D, I745B, I744C, I741B, I745A, I744D, I740B, I741A, I740C, I744E, I740A, I21, I210, I210A, I210B, I211, I211A, I211B, I212, I212A, I212B, I212C, I212E, I212G, I212H, I213, I214, I219, I22, I220, I220A, I220C, I221, I221A, I221B, I228, I228B, I228C, I228F, I228G, I229, I700, I702, I702A, I702B, I708, I708A, I709 |
| Any cancer | C |
| Chronic obstructive pulmonary disease | J42, J429, J429A, J429B, J43, J430, J430A, J431, J431A, J432, J438, J439, J439A, J44, J440, J441, J448, J448A, J448B, J448C, J449 |
| Ischemic heart disease | I21, I210, I210A, I210B, I211, I211A, I211B, I212, I212A, I212B, I212C, I212E, I212G, I212H, I213, I214, I219, I22, I220, I220A, I220C, I221, I221A, I221B, I228, I228B, I228C, I228F, I228G, I229, I23, I230, I231, I232, I233, I234, I235, I236, I236A, I236B, I238, I238A, I24, I240, I240A, I241, I241A, I248, I248A, I249, I25, I250, I251, I251A, I251B, I251C, I252, I252A, I252B, I252C, I253, I254, I254A, I255, I256, I256A, I258, I259 |
| Alcohol abuse | E244, E52, F1, G312, G621, G721, I426, K292, K70, K860, L278A, N979, N980, O354, T51, Z714, Z721. |
| Bleeding | I60-I62, I690-I692, J942, K250, K254, K260, K264, K270, K280, K920-K922, N02, R02, R31, S064, S065 S066. K250, K252, K256, K260, K262, K266, K270, K272, K276, K280, K282, K286, K290, K298A |
| ATC codes | |
| Diabetes | A10 |
| Apixaban | B01AF02 |
| Rivaroxaban | B01AF01. |
| Dabigatran | B01AE07 |
| Vitamin K antagonist | B01AA03, B01AA04. |
| Aspirin (acetylsalicylic acid) | B01AC06, N02BA1 |
| ADP receptor inhibitors | B01AC04, B01AC22, B01AC24 |
| Persantine | B01AC07 |
| NSAID | M01AB, M01AE01, M01AH01, M01AH02, M01AE02 |
| Renin angiotensin system inhibitor | C09AA,C09BA,C09BB,C09CA,C09DA,C09DB,C09XA02  ,C09XA52 |
| Vasodilators | C02DB, C02DD, C02DG |
| Diuretics (not-loop) | C02L, C03A, C03B, C03D, C03E, C03X, C07B, C07D, C08G, C02DA, C09BA, C09DA |
| Beta blockers | C07A,C07B,C07C,C07D,C07F |
| Loop diuretics | C03C |
| CCB | C08,C09BB,C09DB |
| Antiadrenergic agents | C02A, C02B, C02C |
| Digoxin | C01A |
| Amiodaron | C01BD01 |
| Alcohol | N07BB |
| Nordic procedure codes (NCSP) | |
| Ablation for atrial flutter | BFFB03 |
| Ablation for atrial fibrillation | BFFB04 |
| Coronary artery bypass grafting | KFNA00, KFNA10, KFNA20, KFNA96, KFNB00, KFNB96, KFNC10, KFNC20, KFNC30, KFNC40, KFNC50, KFNC60, KFNC96, KFND10, KFND20, KFND96, KFNE00, KFNE10, KFNE20, KFNE96 |
| Percutaneous coronary intervention | KFNG00, KFNG00A, KFNG00B, KFNG00C, KFNG02, KFNG02A, KFNG05, KFNG05A, KFNG10, KFNG12, KFNG20, KFNG30, KFNG40, KFNG96 |
| Prosthetic Valve Replacement | KFKD, KFKH, KFMD, KFMH, KFGE, KFJF,KFMA20 |
